# Ceftibuten-Ledaborbactam Activity against Multidrug-Resistant and Extended-Spectrum-β-Lactamase-Positive Clinical Isolates of *Enterobacterales* from a 2018–2020 Global Surveillance Collection

**DOI:** 10.1128/aac.00934-22

**Published:** 2022-10-26

**Authors:** James A. Karlowsky, Mark G. Wise, Meredith A. Hackel, Daniel C. Pevear, Greg Moeck, Daniel F. Sahm

**Affiliations:** a IHMA, Schaumburg, Illinois, USA; b Department of Medical Microbiology and Infectious Diseases, Max Rady College of Medicine, University of Manitoba, Winnipeg, Manitoba, Canada; c Venatorx Pharmaceuticals, Inc., Malvern, Pennsylvania, USA

**Keywords:** ledaborbactam, VNRX-5236, VNRX-7145, ceftibuten, urinary tract infection, oral therapy, *Enterobacterales*

## Abstract

Ceftibuten-ledaborbactam etzadroxil is a cephalosporin-boronate β-lactamase inhibitor prodrug combination under development as an oral treatment for complicated urinary tract infections caused by multidrug-resistant (MDR) *Enterobacterales* producing serine β-lactamases (Ambler class A, C, and D). *In vivo*, ledaborbactam etzadroxil (formerly VNRX-7145) is cleaved to the active inhibitor ledaborbactam (formerly VNRX-5236). To more completely define the breadth of ceftibuten-ledaborbactam’s activity against important antimicrobial-resistant pathogens, we assessed its *in vitro* activity against phenotypic and genotypic subsets from a 2018–2020 global culture collection of 3,889 clinical isolates of *Enterobacterales*, including MDR organisms, extended-spectrum-β-lactamase (ESBL)-positive organisms, and organisms that are nonsusceptible and resistant to other antimicrobials. MICs were determined by CLSI broth microdilution and interpreted using both CLSI and EUCAST breakpoints. Ledaborbactam was tested at a fixed concentration of 4 μg/mL. β-Lactamase genes were characterized by PCR followed by Sanger sequencing or whole-genome sequencing for selected β-lactam-resistant isolate subsets. At ≤1 μg/mL, ceftibuten-ledaborbactam (MIC_90_, 0.25 μg/mL) inhibited 89.7% of MDR isolates, 98.3% of isolates with a presumptive ESBL-positive phenotype, and 92.6% of trimethoprim-sulfamethoxazole-nonsusceptible, 91.7% of levofloxacin-nonsusceptible, 88.1% of amoxicillin-clavulanate-nonsusceptible, 85.7% of ceftibuten-resistant (MIC >1 μg/mL), and 54.1% of carbapenem-nonsusceptible isolates. Against specific ESBL genotype-positive isolates (AmpC negative, serine carbapenemase negative, and metallo-β-lactamase negative), ceftibuten-ledaborbactam inhibited 96.3% of CTX-M-9 group (MIC_90_, 0.25 μg/mL), 91.5% of CTX-M-1 group (MIC_90_, 0.5 μg/mL), and 88.2% of SHV-positive (MIC_90_, 2 μg/mL) isolates at ≤1 μg/mL. Against specific serine carbapenemase genotype-positive isolates, ceftibuten-ledaborbactam inhibited 85.9% of KPC-positive (MIC_90_, 2 μg/mL) and 82.9% of OXA-48-group-positive (MIC_90_, 2 μg/mL) isolates at ≤1 μg/mL. Continued development of ceftibuten-ledaborbactam appears warranted.

## INTRODUCTION

*Enterobacterales* are the most frequent etiologic agents of uncomplicated and complicated urinary tract infections. β-Lactams are widely prescribed to patients to treat a multitude of infections both in the community and in hospitals, because they provide reliable bactericidal activity and a favorable safety profile. Regrettably, the spread of extended-spectrum β-lactamases (ESBLs), plasmid-mediated AmpCs, serine carbapenemases, and metallo-β-lactamases undermines the efficacy of β-lactams against Gram-negative pathogens (1–3). Combining a novel β-lactamase inhibitor with an approved β-lactam, to prevent its hydrolysis, is a proven strategy to address the treatment challenges associated with evolving and proliferating β-lactamase-producing organisms (4, 5). In contrast to the new parenteral β-lactam–β-lactamase inhibitor combinations that have been introduced into clinical use in the last decade (ceftazidime-avibactam, imipenem-relebactam, meropenem-vaborbactam, and ceftolozane-tazobactam) (4, 5), a new orally bioavailable β-lactam–β-lactamase inhibitor combination has not been approved since amoxicillin-clavulanate in the 1980s. Developing new oral antimicrobial agents to treat outpatients with urinary tract infections caused by *Enterobacterales* carrying ESBLs, AmpC enzymes, and serine carbapenemases constitutes an important unmet medical need (6).

Ledaborbactam (formerly VNRX-5236) is a broad-spectrum boronic acid β-lactamase inhibitor, and its orally bioavailable prodrug, ledaborbactam etzadroxil (formerly VNRX-7145), is being developed in combination with ceftibuten, an oral third-generation cephalosporin, as a potential oral treatment for complicated urinary tract infections caused by serine β-lactamase-producing and multidrug-resistant (MDR) *Enterobacterales*. Ceftibuten-ledaborbactam etzadroxil successfully completed a single- and multiple-ascending-dose phase 1 clinical trial in humans to evaluate its safety and pharmacokinetics (ClinicalTrials.gov identifier NCT04243863). A phase 1 drug-drug interaction study to assess the safety and pharmacokinetics of single and multiple doses of ledaborbactam etzadroxil coadministered with ceftibuten was also recently completed (ClinicalTrials.gov identifier NCT04877379). *In vivo*, the prodrug ledaborbactam etzadroxil undergoes rapid and extensive biotransformation to the active β-lactamase inhibitor ledaborbactam (7–9). Ledaborbactam covalently and reversibly binds and inhibits the active site serine of Ambler class A, C, and D β-lactamases (7–10). Ceftibuten-ledaborbactam has shown potent inhibitory activity against challenge sets of MDR *Enterobacterales* expressing serine β-lactamases (Ambler class A, C, and D), including those that hydrolyze carbapenems such as KPCs and OXAs (11–13). Ledaborbactam alone lacks antibacterial activity (11).

To more completely define the breadth of ceftibuten-ledaborbactam’s activity against important antimicrobial-resistant pathogens, we tested ceftibuten-ledaborbactam and 13 comparators against 3,889 clinical isolates of *Enterobacterales* from a 2018–2020 prevalence-based global surveillance culture collection maintained by IHMA (Schaumburg, IL). We focused our analysis on the ability of ledaborbactam to restore the activity of ceftibuten against isolates with MDR, ESBL-positive, and other antimicrobial-nonsusceptible and -resistant phenotypes and genotypes.

## RESULTS

Isolates were defined as MDR if they tested as nonsusceptible (intermediate or resistant) to at least one agent in three or more antimicrobial categories (14), based on 2022 CLSI M100 MIC breakpoints (15). MDR phenotypes were identified in 31.3% (1,219/3,889) of *Enterobacterales* isolates tested. At ≤1 and ≤0.5 μg/mL, ceftibuten-ledaborbactam inhibited 89.7% and 86.3% of MDR isolates, respectively (Table 1). These results were comparable to those for imipenem-relebactam (86.5% susceptible at the CLSI breakpoint and 91.9% susceptible at the EUCAST breakpoint). Meropenem (84.6% susceptible [CLSI] and 86.5% susceptible [EUCAST]) and imipenem (77.6% and 85.2%) were slightly less active than ceftibuten-ledaborbactam against MDR isolates; 72.0% of MDR isolates were susceptible to tebipenem at its provisional susceptible breakpoint of ≤0.12 μg/mL (16). Using CLSI investigational MIC breakpoints for ceftibuten (susceptible, ≤8 μg/mL; intermediate 16 μg/mL; resistant, ≥32 μg/mL) (15), ceftibuten alone was 25 to 30% less active (reflecting less susceptibility) than ceftibuten-ledaborbactam against MDR isolates. Using EUCAST breakpoints for ceftibuten (susceptible, ≤1 μg/mL; resistant, >1 μg/mL) (17), ceftibuten alone was 55 to 60% less active than ceftibuten-ledaborbactam against MDR isolates. Susceptibility values for MDR isolates for all other agents tested were approximately 50% (67.8% nitrofurantoin susceptible by the EUCAST breakpoint) or less. The ceftibuten-ledaborbactam MIC_90_ was 2 μg/mL for MDR isolates; MIC_90_s were greater than the highest concentration tested for all other agents tested except imipenem-relebactam (2 μg/mL), imipenem (8 μg/mL), and meropenem (16 μg/mL).

**TABLE 1:** *In vitro* activities of ceftibuten-ledaborbactam and comparator agents against MDR, ESBL, and other defined phenotypes of *Enterobacterales* collected from 2018 to 2020

		MIC (μg/mL)			% with MIC interpretation				
					CLSI			EUCAST	
Phenotype^*a*^ (*n*)	Antimicrobial agent	50%	90%	Range	Susceptible	Intermediate	Resistant	Susceptible	Resistant
MDR (1,219)	Ceftibuten-ledaborbactam^*b*^^,^^*c*^	0.12	2	≤0.016 to >32	86.3/89.7	NA^*d*^	13.7/10.3	86.3/89.7	13.7/10.3
	Ceftibuten^*e*^	8	>32	≤0.06 to >32	60.8	11.2	28.0	29.8	70.2
	Amoxicillin-clavulanate^*f*^	16	>32	≤2 to >32	48.2	20.2	31.7	NA	NA
	Cefazolin^*g*^	>32	>32	1 to >32	1.7	5.7	92.6	UTD^*h*^	92.6
	Cefepime^*i*^	16	>16	≤0.25 to >16	34.6	14.8	50.6	31.5	60.0
	Cefixime	>8	>8	≤0.06 to >8	21.5	3.0	75.6	21.5	78.5
	Ceftazidime	>16	>16	0.06 to >16	29.9	6.4	63.7	22.7	70.1
	Imipenem-relebactam	0.12	2	≤0.03 to >8	86.5	5.4	8.1	91.9	8.1
	Imipenem	0.25	8	0.06 to >16	77.6	7.5	14.8	85.2	11.7
	Levofloxacin	8	>8	0.016 to >8	23.9	10.4	65.7	23.9	65.7
	Meropenem	0.06	16	≤0.004 to >64	84.6	2.0	13.5	86.5	10.1
	Nitrofurantoin	32	>128	≤2 to >128	50.9	17.0	32.2	67.8	32.2
	Tebipenem^*j*^	0.06	>4	0.016 to >4	72.0	NA	28.0	72.0	28.0
	Trimethoprim-sulfamethoxazole	>4	>4	≤0.25 to >4	25.9	NA	74.1	25.9	71.9

ESBL^*k*^ (710)	Ceftibuten-ledaborbactam	0.06	0.25	≤0.016 to >32	97.7/98.3	NA	2.3/1.7	97.7/98.3	2.3/1.7
	Ceftibuten	8	>32	≤0.06 to >32	63.4	16.1	20.6	17.0	83.0
	Amoxicillin-clavulanate	8	16	≤2 to >32	65.2	25.9	8.9	NA	NA
	Cefazolin	>32	>32	1 to >32	1.0	2.1	96.9	UTD	96.9
	Cefepime	16	>16	≤0.25 to >16	19.4	19.6	61.0	16.8	74.5
	Cefixime	>8	>8	≤0.06 to >8	8.5	1.5	90.0	8.5	91.5
	Ceftazidime	>16	>16	2 to >16	15.6	9.9	74.5	0	84.4
	Imipenem-relebactam	0.12	0.25	≤0.03 to >8	97.2	2.1	0.7	99.3	0.7
	Imipenem	0.12	0.5	0.06 to 16	96.5	2.3	1.3	98.7	0.3
	Levofloxacin	8	>8	0.016 to >8	26.9	9.3	63.8	26.9	63.8
	Meropenem	0.06	0.12	≤0.004 to 1	100	0	0	100	0
	Nitrofurantoin	32	>128	≤2 to >128	60.0	16.1	23.9	76.1	23.9
	Tebipenem	0.03	0.12	0.016 to >4	92.4	NA	7.6	92.4	7.6
	Trimethoprim-sulfamethoxazole	>4	>4	≤0.25 to >4	32.8	NA	67.2	32.8	67.2

Amoxicillin-clavulanate nonsusceptible (1,309)	Ceftibuten-ledaborbactam	0.06	2	≤0.016 to >32	84.0/88.1	NA	16.0/11.9	84.0/88.1	16.0/11.9
	Ceftibuten	2	>32	≤0.06 to >32	64.2	6.4	29.4	46.8	53.2
	Amoxicillin-clavulanate	32	>32	16 to >32	0	32.6	67.4	NA	NA
	Cefazolin	>32	>32	≤0.5 to >32	1.1	0.8	98.0	UTD	98.0
	Cefepime	≤0.25	>16	≤0.25 to >16	67.8	5.7	26.4	65.2	29.3
	Cefixime	4	>8	≤0.06 to >8	34.1	9.0	56.9	34.1	65.9
	Ceftazidime	2	>16	0.06 to >16	55.8	2.8	41.3	47.9	44.2
	Imipenem-relebactam	0.25	2	≤0.03 to >8	85.3	7.6	7.2	92.8	7.2
	Imipenem	0.5	8	0.06 to >16	74.9	10.0	15.0	85.0	10.8
	Levofloxacin	0.25	>8	0.016 to >8	61.7	6.0	32.3	61.7	32.3
	Meropenem	0.12	8	≤0.004 to >64	86.1	1.5	12.4	87.7	9.4
	Nitrofurantoin	64	>128	≤2 to >128	32.4	26.6	41.0	59.1	40.9
	Tebipenem	0.12	>4	≤0.016 to >4	67.1	NA	32.9	67.1	32.9
	Trimethoprim-sulfamethoxazole	≤0.25	>4	≤0.25 to >4	62.7	NA	37.3	62.6	35.8

Trimethoprim-sulfamethoxazole nonsusceptible (1,258)	Ceftibuten-ledaborbactam	0.06	0.5	≤0.016 to >32	90.2/92.6	NA	9.8/7.4	90.2/92.6	9.8/7.4
	Ceftibuten	2	>32	≤0.06 to >32	71.8	8.2	20.0	45.6	54.4
	Amoxicillin-clavulanate	8	>32	≤2 to >32	61.1	14.7	24.2	NA	NA
	Cefazolin	>32	>32	≤0.5 to >32	17.2	8.3	74.4	UTD	74.4
	Cefepime	2	>16	≤0.25 to >16	50.9	10.6	38.6	49.1	45.2
	Cefixime	>8	>8	≤0.06 to >8	38.2	2.9	58.8	38.2	61.8
	Ceftazidime	8	>16	≤0.03 to >16	49.8	4.5	45.7	44.3	50.2
	Imipenem-relebactam	0.12	2	≤0.03 to >8	88.5	4.8	6.8	93.2	6.8
	Imipenem	0.25	4	0.06 to >16	82.0	5.7	12.2	87.8	9.7
	Levofloxacin	2	>8	0.016 to >8	37.7	9.1	53.2	37.7	53.2
	Meropenem	0.06	4	≤0.004 to >64	87.8	1.4	10.7	89.3	8.6
	Nitrofurantoin	32	>128	≤2 to >128	53.7	15.9	30.4	69.6	30.4
	Tebipenem	0.03	>4	≤0.016 to >4	78.5	NA	21.5	78.5	21.5
	Trimethoprim-sulfamethoxazole	>4	>4	4 to >4	0	NA	100	0	96.6

Levofloxacin nonsusceptible (1,142)	Ceftibuten-ledaborbactam	0.06	1	≤0.016 to >32	88.8/91.7	NA	11.2/8.3	88.8/91.7	11.2/8.3
	Ceftibuten	4	>32	≤0.06 to >32	65.9	10.0	24.1	38.5	61.5
	Amoxicillin-clavulanate	8	32	≤2 to >32	56.1	16.6	27.2	NA	NA
	Cefazolin	>32	>32	≤0.5 to >32	13.1	6.8	80.0	UTD	80.0
	Cefepime	8	>16	≤0.25 to >16	41.4	11.7	46.8	38.8	54.1
	Cefixime	>8	>8	≤0.06 to >8	30.9	3.6	65.5	30.9	69.1
	Ceftazidime	16	>16	0.06 to >16	39.1	5.0	55.9	32.7	60.9
	Imipenem-relebactam	0.12	2	≤0.03 to >8	86.8	5.8	7.4	92.6	7.4
	Imipenem	0.25	8	0.06 to >16	79.9	6.4	13.7	86.3	11.0
	Levofloxacin	>8	>8	1 to >8	0	15.2	84.8	0	84.8
	Meropenem	0.06	16	≤0.004 to >64	85.6	1.7	12.7	87.3	10.1
	Nitrofurantoin	32	>128	≤2 to >128	50.4	14.4	35.2	64.8	35.2
	Tebipenem	0.06	>4	0.008 to >4	73.6	NA	26.4	73.6	26.4
	Trimethoprim-sulfamethoxazole	>4	>4	≤0.25 to >4	31.3	NA	68.7	31.3	68.7

Tebipenem resistant (553)	Ceftibuten-ledaborbactam	0.12	>32	≤0.016 to >32	70.9/77.2	NA	29.1/22.8	70.9/77.2	29.1/22.8
	Ceftibuten	8	>32	≤0.06 to >32	53.9	5.4	40.7	41.6	58.4
	Amoxicillin-clavulanate	32	>32	≤2 to >32	22.2	6.7	71.1	NA	NA
	Cefazolin	>32	>32	≤0.5 to >32	4.2	7.6	88.3	UTD	88.2
	Cefepime	1	>16	≤0.25 to >16	52.8	7.6	39.6	51.0	43.4
	Cefixime	>8	>8	≤0.06 to >8	32.4	2.9	64.7	32.4	67.6
	Ceftazidime	>16	>16	≤0.03 to >16	43.6	2.7	53.7	39.8	56.4
	Imipenem-relebactam	1	>8	0.06 to >8	51.0	26.0	23.0	77.0	23.0
	Imipenem	2	>16	0.06 to >16	32.5	27.1	40.3	59.7	26.0
	Levofloxacin	1	>8	0.016 to >8	45.4	7.2	47.4	45.5	47.4
	Meropenem	0.25	64	≤0.004 to >64	66.4	4.3	29.3	70.7	22.1
	Nitrofurantoin	128	>128	≤2 to >128	15.6	23.9	60.6	39.4	60.6
	Tebipenem	0.5	>4	0.25 to >4	0	NA	100	0	100
	Trimethoprim-sulfamethoxazole	2	>4	≤0.25 to >4	51.2	NA	48.8	51.2	46.5

Carbapenem nonsusceptible (246)	Ceftibuten-ledaborbactam	0.5	>32	≤0.016 to >32	54.1/63.8	NA	45.9/36.2	54.1/63.8	45.9/36.2
	Ceftibuten	32	>32	≤0.06 to >32	34.8	8.9	56.3	18.7	81.3
	Amoxicillin-clavulanate	>32	>32	≤2 to >32	4.9	6.9	88.2	NA	NA
	Cefazolin	>32	>32	1 to >32	1.2	0	98.8	UTD	98.8
	Cefepime	>16	>16	≤0.25 to >16	22.8	4.1	73.2	20.7	76.8
	Cefixime	>8	>8	≤0.06 to >8	13.8	2.8	83.3	13.8	86.2
	Ceftazidime	>16	>16	0.06 to >16	19.1	2.4	78.5	17.5	80.9
	Imipenem-relebactam	1	>8	0.06 to >8	53.7	13.0	33.3	66.7	33.3
	Imipenem	8	>16	0.12 to >16	8.9	27.2	63.8	36.2	56.5
	Levofloxacin	>8	>8	0.03 to >8	27.2	7.7	65.0	27.2	65.0
	Meropenem	8	>64	0.03 to >64	23.6	9.8	66.7	33.3	50.0
	Nitrofurantoin	128	>128	8 to >128	24.0	15.9	60.2	39.8	60.2
	Tebipenem	>4	>4	0.03 to >4	11.4	NA	88.6	11.4	88.6
	Trimethoprim-sulfamethoxazole	>4	>4	≤0.25 to >4	30.9	NA	69.1	33.3	66.7

a All phenotypes were defined using CLSI M100 MIC breakpoints (15).

b Ledaborbactam was tested at a fixed concentration of 4 μg/mL in combination with doubling dilutions of ceftibuten.

c Provisional susceptible MIC breakpoints of ≤0.5 μg/mL/≤1 μg/mL and provisional resistant MIC breakpoints of ≥1 μg/mL/2 μg/mL were applied for ceftibuten-ledaborbactam.

d NA, not applicable.

e CLSI publishes investigational MIC breakpoints for ceftibuten (susceptible, ≤8 μg/mL; intermediate, 16 μg/mL; resistant, ≥32 μg/mL) for testing and reporting of urinary tract isolates only (15). EUCAST publishes MIC breakpoints for ceftibuten (susceptible, ≤1 μg/mL; resistant, >1 μg/mL) for infections originating from the urinary tract (17).

f Amoxicillin-clavulanate was tested in a 2:1 ratio (15), and therefore, MICs could not be interpreted using EUCAST breakpoints, which require clavulanate to be tested at a fixed concentration of 2 μg/mL (17).

g For cefazolin MICs, the CLSI parenteral breakpoints were used (susceptible, ≤2 μg/mL; intermediate, 4 μg/mL; resistant, ≥8 μg/mL).

h UTD, unable to determine because the cefazolin concentration range tested did not encompass the susceptible breakpoint.

i CLSI does not define an intermediate MIC breakpoint category for cefepime tested against *Enterobacterales*. In its place, CLSI publishes a “susceptible–dose-dependent” breakpoint category (MIC, 4 to 8 μg/mL). For cefepime, percentages in the intermediate column are isolates that tested with a susceptible–dose-dependent MIC (4 to 8 μg/mL).

j A provisional susceptibility breakpoint of ≤0.12 μg/mL was applied for tebipenem (16).

k ESBL phenotype isolates were defined as isolates of E. coli, K. pneumoniae, K. oxytoca, and P. mirabilis with ceftazidime MICs of ≥2 μg/mL and meropenem MICs of ≤1 μg/mL.

Overall, 24.6% (710/2,887) of Escherichia coli, Klebsiella pneumoniae, Klebsiella oxytoca, and Proteus mirabilis isolates demonstrated presumptive ESBL phenotypes (ceftazidime MICs of ≥2 μg/mL and meropenem MICs of ≤1 μg/mL). Against presumptive ESBL-positive isolates of E. coli, K. pneumoniae, K. oxytoca, and P. mirabilis, the ceftibuten-ledaborbactam MIC_90_ was 0.25 μg/mL; 98.3% and 97.7% of presumptive ESBL-positive isolates were inhibited by ceftibuten-ledaborbactam at ≤1 and ≤0.5 μg/mL, respectively. Of the 710 isolates with presumptive ESBL phenotypes (ceftazidime MICs of ≥2 μg/mL and meropenem MICs of ≤1 μg/mL), 194 were subjected to either whole-genome sequencing or PCR followed by Sanger sequencing for β-lactamase genes based upon selection algorithms. Of these 194 isolates, 155 (80%) carried one or more ESBL genes that presumptively would account for the observed phenotype. Imipenem-relebactam (97.2% susceptible at the CLSI breakpoint and 99.3% susceptible at the EUCAST breakpoint) demonstrated *in vitro* activity similar to ceftibuten-ledaborbactam against presumptive ESBL-positive isolates. Presumptive ESBL-positive isolates were >20% less susceptible to nitrofurantoin (60.0% susceptible at the CLSI breakpoint and 76.1% susceptible at the EUCAST breakpoint), >30% less susceptible to amoxicillin-clavulanate (65.2% susceptible), and >65% less susceptible to both trimethoprim-sulfamethoxazole and levofloxacin than to ceftibuten-ledaborbactam.

At ≤1 μg/mL, ceftibuten-ledaborbactam also inhibited 92.6% of trimethoprim-sulfamethoxazole-nonsusceptible, 91.7% of levofloxacin-nonsusceptible, and 88.1% of amoxicillin-clavulanate-nonsusceptible isolates. Against tebipenem-resistant isolates, MIC_90_s for all antimicrobial agents were greater than the highest concentration tested except for meropenem (MIC_90_, 64 μg/mL). Ceftibuten-ledaborbactam inhibited 77.3% of tebipenem-resistant isolates at ≤1 μg/mL and 70.9% at ≤0.5 μg/mL, which were higher percentages than for all comparators. The ceftibuten-ledaborbactam MIC_90_ was >32 μg/mL against carbapenem-nonsusceptible isolates; 63.8% and 54.1% of isolates were inhibited at ceftibuten-ledaborbactam concentrations of ≤1 and ≤0.5 μg/mL, respectively, and both of these percentages were higher than those of all other comparator agents, including the most active agent administered intravenously only, imipenem-relebactam (53.7% susceptible).

Table 2 presents the *in vitro* activity of ceftibuten-ledaborbactam against *Enterobacterales* isolates with defined β-lactamase genotypes. Against ESBL-positive isolates (AmpC negative, serine carbapenemase negative, metallo-β-lactamase [MBL] negative), ceftibuten-ledaborbactam inhibited 96.3% of CTX-M-9 group (MIC_90_, 0.25 μg/mL), 91.5% of CTX-M-1 group (MIC_90_, 0.5 μg/mL), and 88.2% of SHV-positive (MIC_90_, 2 μg/mL) isolates at ≤1 μg/mL. Among the 99 serine carbapenemase-positive, MBL-negative isolates, 85.9% of KPC-positive (MIC_90_, 2 μg/mL) and 82.9% of OXA-48-group-positive (MIC_90_, 2 μg/mL) isolates were inhibited by ceftibuten-ledaborbactam at ≤1 μg/mL. Sixty-five MBL-positive isolates were identified. As expected, none of the agents tested in this study were active against the MBL producers.

**TABLE 2:** *In vitro* activities of ceftibuten-ledaborbactam and comparator agents against all global isolates and defined genotypes of *Enterobacterales* collected from 2018 to 2020

Genotype^*a*^ (*n*)	Antimicrobial agent	MIC (μg/mL)			% with MIC interpretation				
					CLSI			EUCAST	
		50%	90%	Range	Susceptible	Intermediate	Resistant	Susceptible	Resistant
CTX-M-1 group (129)	Ceftibuten-ledaborbactam^*b*^^,^^*c*^	0.06	0.5	≤0.016 to >32	90.7/91.5	NA^*d*^	9.3/8.5	90.7/91.5	9.3/8.5
	Ceftibuten^*e*^	8	>32	≤0.06 to >32	54.3	22.5	23.3	4.7	95.3
	Amoxicillin-clavulanate^*f*^	8	16	≤2 to >32	65.9	28.7	5.4	NA	NA
	Cefazolin^*g*^	>32	>32	32 to >32	0.0	0	100	0	100
	Cefepime^*h*^	>16	>16	1 to >16	2.3	16.3	81.4	1.6	93.8
	Cefixime	>8	>8	≤0.06 to >8	0.8	0.8	98.4	0.8	99.2
	Ceftazidime	>16	>16	4 to >16	4.7	7.8	87.6	0	95.3
	Imipenem-relebactam	0.12	0.5	0.06 to 1	100.0	0	0	100	0
	Imipenem	0.12	0.5	0.06 to 2	98.4	1.6	0	100	0
	Levofloxacin	8	>8	0.03 to >8	18.6	10.1	71.3	18.6	71.3
	Meropenem	0.06	0.12	0.016 to 8	92.2	2.3	5.4	94.6	0
	Nitrofurantoin	64	>128	4 to >128	48.1	17.1	34.9	65.1	34.9
	Tebipenem^*i*^	0.03	0.12	0.016 to >4	91.5	NA	8.5	91.5	8.5
	Trimethoprim-sulfamethoxazole	>4	>4	≤0.25 to >4	20.2	NA	79.8	20.2	79.1

CTX-M-9 group (27)	Ceftibuten-ledaborbactam	0.12	0.25	0.03 to 8	96.3/96.3	NA	3.7/3.7	96.3/96.3	3.7/3.7
	Ceftibuten	4	>32	0.25 to >32	81.5	7.4	11.1	14.8	85.2
	Amoxicillin-clavulanate	4	8	≤2 to >32	92.6	0	7.4	NA	NA
	Cefazolin	>32	>32	32 to >32	0	0	100	0	100
	Cefepime	16	>16	2 to >16	3.7	40.7	55.6	0	74.1
	Cefixime	>8	>8	2 to >8	0	3.7	96.3	0	100
	Ceftazidime	16	>16	1 to >16	18.5	29.6	51.9	7.4	81.5
	Imipenem-relebactam	0.12	0.25	0.06 to 1	100	0	0	100	0
	Imipenem	0.12	0.25	0.06 to 2	96.3	3.7	0	100	0
	Levofloxacin	>8	>8	0.03 to >8	11.1	0	88.9	11.1	88.9
	Meropenem	0.06	0.12	0.016 to 0.12	100	0	0	100	0
	Nitrofurantoin	8	64	≤2 to >128	88.9	7.4	3.7	96.3	3.7
	Tebipenem	0.03	0.12	0.016 to 0.25	96.3	NA	3.7	96.3	3.7
	Trimethoprim-sulfamethoxazole	>4	>4	≤0.25 to >4	25.9	NA	74.1	25.9	74.1

SHV-ESBL (17)	Ceftibuten-ledaborbactam	0.06	2	≤0.016 to 2	88.2/88.2	NA	11.8/11.8	88.2/88.2	11.8/11.8
	Ceftibuten	8	>32	0.25 to >32	58.8	17.6	23.5	23.5	76.5
	Amoxicillin-clavulanate	8	32	≤2 to >32	70.6	17.6	11.8	NA	NA
	Cefazolin	>32	>32	16 to >32	0	0	100	0	100
	Cefepime	16	>16	≤0.25 to >16	35.3	11.8	52.9	29.4	64.7
	Cefixime	>8	>8	8 to >8	0	0	100	0	100
	Ceftazidime	>16	>16	8 to >16	0	11.8	88.2	0	100
	Imipenem-relebactam	0.12	1	0.12 to 1	100	0	0	100	0
	Imipenem	0.25	1	0.12 to 2	94.1	5.9	0	100	0
	Levofloxacin	4	>8	0.06 to >8	29.4	11.8	58.8	29.4	58.8
	Meropenem	0.06	0.25	0.016 to 4	94.1	0	5.9	94.1	0
	Nitrofurantoin	64	>128	8 to >128	35.3	23.5	41.2	58.8	41.2
	Tebipenem	0.06	0.5	0.016 to >4	76.5	NA	23.5	76.5	23.5
	Trimethoprim-sulfamethoxazole	>4	>4	≤0.25 to >4	11.8	NA	88.2	11.8	82.4

KPC (64)	Ceftibuten-ledaborbactam	0.25	2	≤0.016 to 32	68.8/85.9	NA	31.3/14.1	68.8/85.9	31.3/14.1
	Ceftibuten	16	>32	0.25 to >32	31.3	25.0	43.8	6.3	93.8
	Amoxicillin-clavulanate	>32	>32	16 to >32	0	7.8	92.2	NA	NA
	Cefazolin	>32	>32	>32	0	0	100	0	100
	Cefepime	>16	>16	1 to >16	4.7	4.7	90.6	1.6	93.8
	Cefixime	>8	>8	1 to >8	1.6	1.6	96.9	1.6	98.4
	Ceftazidime	>16	>16	8 to >16	0	3.1	96.9	0	100
	Imipenem-relebactam	0.25	1	0.06 to >8	90.6	4.7	4.7	95.3	4.7
	Imipenem	16	>16	0.12 to >16	3.1	3.1	93.8	6.3	90.6
	Levofloxacin	>8	>8	0.03 to >8	14.1	1.6	84.4	14.1	84.4
	Meropenem	32	>64	2 to >64	0	3.1	96.9	3.1	78.1
	Nitrofurantoin	>128	>128	8 to >128	12.5	7.8	79.7	20.3	79.7
	Tebipenem	>4	>4	0.25 to >4	0	NA	100	0	100
	Trimethoprim-sulfamethoxazole	>4	>4	≤0.25 to >4	17.2	NA	82.8	17.2	79.7

OXA-48-group (35)	Ceftibuten-ledaborbactam	0.25	2	0.03 to 32	60.0/82.9	NA	40.0/17.1	60.0/82.9	40.0/17.1
	Ceftibuten	>32	>32	0.12 to >32	28.6	5.7	65.7	8.6	91.4
	Amoxicillin-clavulanate	>32	>32	32 to >32	0	0	100	NA	NA
	Cefazolin	>32	>32	32 to >32	0	0	100	0	100
	Cefepime	>16	>16	≤0.25 to >16	5.7	5.7	88.6	5.7	94.3
	Cefixime	>8	>8	0.25 to >8	8.6	0	91.4	8.6	91.4
	Ceftazidime	>16	>16	0.5 to >16	5.7	0	94.3	2.9	94.3
	Imipenem-relebactam	2	8	0.25 to >8	28.6	37.1	34.3	65.7	34.3
	Imipenem	4	8	0.25 to 16	14.3	31.4	54.3	45.7	37.1
	Levofloxacin	>8	>8	0.06 to >8	5.7	11.4	82.9	5.7	82.9
	Meropenem	16	64	0.25 to >64	17.1	11.4	71.4	28.9	57.1
	Nitrofurantoin	>128	>128	8 to >128	8.6	25.7	65.7	34.3	65.7
	Tebipenem	>4	>4	0.06 to >4	2.9	NA	97.1	2.9	97.1
	Trimethoprim-sulfamethoxazole	>4	>4	≤0.25 to >4	20.0	NA	80.0	20.0	74.3

a All genotypes were identified using defined using PCR followed by Sanger sequencing or whole-genome sequencing as described in Materials and Methods. ESBL-positive isolates (CTX-M-1 group, CTX-M-9 group, and SHV-ESBL) exclude isolates simultaneously carrying an AmpC-type enzyme, serine carbapenemase, or an MBL. Serine carbapenemase-positive isolates (KPC and OXA-48 group) exclude isolates simultaneously carrying an MBL.

b Ledaborbactam was tested at a fixed concentration of 4 μg/mL in combination with doubling dilutions of ceftibuten.

c A provisional susceptible MIC breakpoints of ≤0.5 μg/mL/≤1 μg/mL and provisional resistant MIC breakpoints of ≥1 μg/mL/2 μg/mL were applied for ceftibuten-ledaborbactam.

d NA, not applicable.

e CLSI publishes investigational MIC breakpoints for ceftibuten (susceptible, ≤8 μg/mL; intermediate, 16 μg/mL; resistant, ≥32 μg/mL) for testing and reporting of urinary tract isolates only (15). EUCAST publishes MIC breakpoints for ceftibuten (susceptible, ≤1 μg/mL; resistant, >1 μg/mL) for infections originating in the urinary tract (17).

f Amoxicillin-clavulanate was tested in a 2:1 ratio (15), and therefore, MICs could not be interpreted using EUCAST breakpoints, which require clavulanate to be tested at a fixed concentration of 2 μg/mL (17).

g For cefazolin MICs, the CLSI parenteral breakpoints were used (susceptible, ≤2 μg/mL; intermediate, 4 μg/mL; resistant, ≥8 μg/mL).

h CLSI does not define an intermediate MIC breakpoint category for cefepime tested against *Enterobacterales*. In its place, CLSI publishes the breakpoint category known as “susceptible–dose-dependent” (MIC, 4 to 8 μg/mL). For cefepime, percentages in the intermediate column are isolates that tested with a susceptible–dose-dependent MIC (4 to 8 μg/mL).

i A provisional susceptible breakpoint of ≤0.12 μg/mL was applied for tebipenem (16).

Table 3 depicts the MIC distributions for ceftibuten-ledaborbactam and ceftibuten against MDR, ESBL, and other phenotypic and genotypic subsets of antimicrobial-nonsusceptible and -resistant clinical isolates of *Enterobacterales*. At a concentration of ≤1 μg/mL, ceftibuten-ledaborbactam inhibited 73.5% of isolates with ceftibuten MICs of >8 μg/mL (ceftibuten nonsusceptible, CLSI definition) (15) and 85.7% of isolates with ceftibuten MICs of >1 μg/mL (ceftibuten resistant, EUCAST definition) (17).

**TABLE 3:** Cumulative frequency distributions of ceftibuten-ledaborbactam^*a*^ and ceftibuten MICs against phenotypic and genotypic subsets of antimicrobial-nonsusceptible and -resistant clinical isolates of *Enterobacterales*

Phenotype or genotype (no. of isolates) and agent	Cumulative percentage of isolates inhibited by MIC (μg/mL) (no. of isolates with MIC)^*b*^												
	≤0.016	0.03	0.06^*c*^	0.12	0.25	0.5	1	2	4	8	16	32	>32
Ceftibuten nonsusceptible, CLSI (597)													
Ceftibuten-ledaborbactam	0.5 (3)	5.0 (27)	18.1 (78)	37.7 (117)	50.9 (79)	65.2 (85)	73.5 (50)	82.1 (51)	84.6 (15)	86.8 (13)	87.3 (3)	89.4 (13)	**100 (63)**
Ceftibuten											25.6 (153)	43.2 (105)	**100 (339)**
Ceftibuten nonsusceptible, EUCAST (1,123)													
Ceftibuten-ledaborbactam	2.0 (22)	15.2 (149)	41.1 (290)	62.9 (245)	72.0 (102)	80.9 (100)	85.7 (54)	**90.5 (54)**	91.8 (15)	93.0 (13)	93.2 (3)	94.4 (13)	100 (63)
Ceftibuten								16.6 (186)	28.9 (138)	46.8 (202)	60.5 (153)	69.8 (105)	**100 (339)**
MDR phenotype (1,219)													
Ceftibuten-ledaborbactam	5.3 (65)	21.2 (194)	49.5 (345)	71.4 (266)	79.9 (104)	86.3 (78)	89.7 (41)	**92.5** (34)	93.0 (7)	93.8 (10)	93.9 (1)	94.9 (12)	100 (62)
Ceftibuten			8.5 (104)	11.6 (38)	16.8 (63)	24.6 (95)	29.8 (63)	37.7 (96)	46.3 (105)	60.8 (177)	72.0 (137)	79.7 (93)	**100 (248)**
ESBL phenotype (710)^*d*^													
Ceftibuten-ledaborbactam	3.4 (24)	21.4 (128)	55.4 (241)	82.7 (194)	**93.1 (74)**	97.7 (33)	98.3 (4)	99.0 (5)	99.2 (1)	99.4 (2)	99.4 (0)	99.9 (3)	100 (1)
Ceftibuten			3.8 (27)	5.2 (10)	8.5 (23)	12.3 (27)	17.0 (34)	28.2 (79)	42.5 (102)	63.4 (148)	79.4 (114)	89.6 (72)	**100 (74)**
Amoxicillin-clavulanate-nonsusceptible (1,309)													
Ceftibuten-ledaborbactam	9.8 (128)	27.8 (237)	52.9 (329)	70.2 (226)	77.0 (89)	84.1 (93)	88.1 (53)	**91.9** (50)	93.1 (15)	94.1 (13)	94.3 (3)	95.2 (12)	100 (63)
Ceftibuten			10.5 (137)	18.8 (110)	25.9 (93)	37.7 (154)	46.9 (121)	54.2 (96)	57.2 (39)	64.2 (92)	70.6 (84)	76.2 (74)	**100 (311)**
Trimethoprim-sulfamethoxazole-nonsusceptible (1,258)													
Ceftibuten-ledaborbactam	7.4 (93)	30.2 (287)	62.0 (400)	78.7 (210)	85.4 (84)	**90.2 (61)**	92.6 (30)	94.4 (23)	94.8 (5)	95.2 (4)	95.2 (1)	95.9 (9)	100 (51)
Ceftibuten			12.2 (154)	21.4 (115)	32.0 (134)	41.0 (113)	45.6 (58)	52.9 (92)	60.4 (94)	71.8 (143)	80.0 (103)	85.7 (72)	**100 (180)**
Levofloxacin-nonsusceptible (1,142)													
Ceftibuten-ledaborbactam	7.6 (87)	22.8 (173)	50.7 (319)	73.1 (256)	82.0 (102)	88.8 (77)	**91.7 (33)**	93.6 (22)	94.0 (4)	94.5 (6)	94.6 (1)	95.4 (9)	100 (53)
Ceftibuten			11.6 (132)	17.3 (65)	25.0 (88)	33.9 (102)	38.5 (53)	45.4 (78)	53.0 (87)	65.9 (148)	75.9 (114)	82.7 (77)	**100 (198)**
Tebipenem-resistant (553)													
Ceftibuten-ledaborbactam	14.8 (82)	32.7 (99)	44.3 (64)	53.2 (49)	60.9 (43)	70.9 (55)	77.2 (35)	82.6 (30)	84.4 (10)	86.4 (11)	87.0 (3)	88.8 (10)	**100 (62)**
Ceftibuten			23.9 (132)	27.8 (22)	32.2 (24)	38.9 (37)	41.6 (15)	45.0 (19)	47.7 (15)	53.9 (34)	59.3 (30)	65.6 (35)	**100 (190)**
Carbapenem-nonsusceptible (246)													
Ceftibuten-ledaborbactam	2.8 (7)	12.6 (24)	26.4 (34)	37.0 (26)	43.1 (15)	54.1 (27)	63.8 (24)	69.5 (14)	69.9 (1)	71.1 (3)	71.5 (1)	75.2 (9)	**100 (61)**
Ceftibuten			3.7 (9)	5.3 (4)	6.9 (4)	12.2 (13)	18.7 (16)	23.2 (11)	27.2 (10)	34.6 (18)	43.5 (22)	51.6 (20)	**100 (119**)
CTX-M-1 group (129)^*e*^													
Ceftibuten-ledaborbactam	5.4 (7)	25.6 (26)	54.3 (37)	81.4 (35)	86.8 (7)	**90.7 (5)**	91.5 (1)	95.3 (5)	95.3 (0)	96.9 (2)	96.9 (0)	98.4 (2)	100 (2)
Ceftibuten			0.8 (1)	0.8 (0)	0.8 (0)	1.6 (1)	4.7 (4)	15.5 (14)	31.0 (20)	54.5 (30)	76.7 (29)	84.5 (10)	**100** (20)
CTX-M-9 group (27)^*e*^													
Ceftibuten-ledaborbactam		3.7 (1)	29.6 (7)	77.8 (13)	**96.3 (5)**	96.3 (0)	96.3 (0)	96.3 (0)	96.3 (0)	100 (1)			
Ceftibuten					3.7 (1)	7.4 (1)	14.8 (2)	37.0 (6)	77.8 (11)	81.5 (1)	88.9 (2)	88.9 (0)	**100** (3)
SHV-ESBL (17)^*e*^													
Ceftibuten-ledaborbactam	5.9 (1)	23.5 (3)	52.9 (5)	64.7 (2)	70.6 (1)	88.2 (3)	88.2 (0)	**100 (2)**					
Ceftibuten					5.9 (1)	5.9 (0)	23.5 (3)	41.2 (3)	41.2 (0)	58.8 (3)	76.5 (3)	82.4 (1)	**100 (3)**
Acquired AmpC (13)^*f*^													
Ceftibuten-ledaborbactam			7.7 (1)	46.2 (5)	53.8 (1)	53.8 (0)	61.5 (1)	76.9 (2)	84.6 (1)	84.6 (0)	84.6 (0)	**100 (2)**	
Ceftibuten												15.4 (2)	**100 (11)**
KPC (64)^*g*^													
Ceftibuten-ledaborbactam	1.6 (1)	7.8 (4)	28.1 (13)	43.8 (10)	50.0 (4)	68.8 (12)	85.9 (11)	**95.3 (6)**	96.9 (1)	98.4 (1)	98.4 (0)	100 (1)	
Ceftibuten					1.6 (1)	4.7 (2)	6.3 (1)	9.4 (2)	18.8 (6)	31.8 (8)	56.3 (16)	73.4 (11)	**100 (17)**
OXA-48-group (35)^*g*^													
Ceftibuten-ledaborbactam		5.7 (2)	20.0 (5)	31.4 (4)	51.4 (7)	60.0 (3)	82.9 (8)	**91.4 (3)**	91.4 (0)	97.1 (2)	97.1 (0)	100 (1)	
Ceftibuten				2.9 (1)	2.9 (0)	8.6 (2)	8.6 (0)	8.6 (0)	14.3 (2)	28.6 (5)	34.3 (2)	45.7 (4)	**100 (19)**

a Ledaborbactam was tested at a fixed concentration of 4 μg/mL in combination with doubling dilutions of ceftibuten.

b Boldface indicates the MIC_90_ for each MIC distribution.

c For ceftibuten, 0.06 μg/mL is ≤0.06 μg/mL.

d ESBL phenotype isolates were defined as isolates of E. coli, K. pneumoniae, K. oxytoca, and P. mirabilis with ceftazidime MICs of ≥2 μg/mL and meropenem MICs of ≤1 μg/mL.

e ESBL-positive isolates exclude isolates simultaneously carrying an AmpC-type enzyme, serine carbapenemase, or a metallo-β-lactamase.

f Acquired AmpC-positive isolates exclude isolates simultaneously carrying an ESBL, serine carbapenemase, or a metallo-β-lactamase.

g Serine carbapenemase-positive isolates exclude isolates simultaneously carrying a metallo-β-lactamase.

Similar to most comparator agents, ceftibuten-ledaborbactam was equally active against *Enterobacterales* regardless of infection source (see Table S1 in the supplemental material), including against the 1,210 urinary tract infection isolates of *Enterobacterales* tested in this study (Table S2). Ceftibuten-ledaborbactam MIC_90_s varied by 8-fold across the *Enterobacterales* species tested (0.06 to 0.5 μg/mL) except for the Enterobacter cloacae complex, which exhibited an MIC_90_ of 4 μg/mL (Table S3). The percentage of isolates inhibited for all taxonomic groups aside from the E. cloacae complex was ≥92.2% at ≤0.5 μg/mL and ≥94.3% at ≤1 μg/mL. For the E. cloacae complex, 82.0% of isolates were inhibited at a ceftibuten-ledaborbactam concentration of ≤1 μg/mL (Table S3). This percentage was similar to the rates of susceptibility to the most active oral agents, levofloxacin (87.8%), trimethoprim-sulfamethoxazole (83.8%), and tebipenem (82.9%) (data not shown).

## DISCUSSION

Ceftibuten in combination with ledaborbactam etzadroxil is under development as an oral treatment for complicated urinary tract infections, including acute pyelonephritis, caused by serine β-lactamase-producing *Enterobacterales* (7), which are identified as CDC and WHO priority pathogens (i.e., carbapenem-resistant and/or third-generation-cephalosporin-resistant *Enterobacterales*) (2, 3). The development of new agents, particularly oral agents, that target CDC and WHO priority pathogens is critical (2, 3), as oral agents promote outpatient treatment, facilitate step-down therapy, and shorten duration of, or prevent, hospitalization. The current study demonstrated that ledaborbactam improved the *in vitro* activity of ceftibuten (lowered its MIC) for the vast majority of MDR and ESBL-positive isolates as well as for other phenotypic and genotypic subsets of antimicrobial-nonsusceptible and -resistant clinical isolates of *Enterobacterales* in a 2018–2020 global collection.

Currently prescribed agents to treat urinary tract infections have a number of shortcomings. Resistance and multidrug resistance to trimethoprim-sulfamethoxazole, fluoroquinolones, and oral β-lactams including amoxicillin-clavulanate is frequently encountered and increasing, while other agents have pharmacokinetic (nitrofurantoin) and spectrum (nitrofurantoin and fosfomycin) limitations (18). New oral agents to treat both complicated and uncomplicated urinary tract infections are urgently needed to address the inadequacies of currently available agents (6, 18). Ceftibuten-ledaborbactam etzadroxil holds promise for the treatment of patients with complicated urinary tract infection where resistant pathogens are suspected or where hospital avoidance and oral therapy are reasonable.

Our study demonstrated that the orally bioavailable ceftibuten-ledaborbactam had greater *in vitro* potency than carbapenems (meropenem and imipenem) and potency similar to that of newer carbapenem-β-lactamase combination (imipenem-relebactam) parenteral therapies against MDR isolates of *Enterobacterales*. For MDR isolates, MIC_90_s were 2 μg/mL for ceftibuten-ledaborbactam and imipenem-relebactam, 8 μg/mL for imipenem, and 16 μg/mL for meropenem; the MIC_90_s for imipenem and imipenem-relebactam may reflect, in part, the inclusion of *Proteeae* in the isolates (8.8%; 107 of the 1,219 MDR isolates) tested (Table 1). Ceftibuten-ledaborbactam at 1 μg/mL inhibited 89.7% of MDR isolates, compared to susceptibilities of 86.5% for imipenem-relebactam, 84.6% for meropenem, and 77.6% for imipenem. Previous studies have also reported that ceftibuten-ledaborbactam demonstrated similar potency *in vitro* compared to carbapenems (meropenem) and current β-lactam/β-lactamase inhibitors (meropenem-vaborbactam and ceftazidime-avibactam) against MDR *Enterobacterales* (12, 19). In one recent study of 205 challenge set isolates of *Enterobacterales*, Mendes et al. reported that ceftibuten-ledaborbactam MICs (MIC_50_, 0.12 μg/mL; MIC_90_, 1 μg/mL) were 2- to 4-fold lower than for ceftazidime-avibactam (MIC_50_, 0.5 μg/mL; MIC_90_, 2 μg/mL) (13).

Our study of recent isolates collected globally demonstrated that ceftibuten-ledaborbactam possesses potent *in vitro* activity (>88% of isolates were inhibited at 1 μg/mL) against ESBL-positive (CTX-M-1 group-, CTX-M-9 group-, and SHV-positive) isolates of *Enterobacterales*, confirming earlier reports (12, 13, 19). For molecularly defined ESBL-positive isolates, ceftibuten-ledaborbactam (MIC_90_, 0.25 to 2 μg/mL), imipenem (MIC_90_, 0.25 to 1 μg/mL), and meropenem (MIC_90_, 0.12 to 0.25 μg/mL) had low MICs (≤1 μg/mL) and comparable *in vitro* activities (Table 2). Similar data were observed for presumptive ESBL phenotypes: ceftibuten-ledaborbactam (MIC_90_, 0.25 μg/mL), imipenem (MIC_90_, 0.5 μg/mL), and meropenem (MIC_90_, 0.12 μg/mL) (Table 1). Chatwin et al. previously reported an MIC_90_ of 0.25 μg/mL for ceftibuten-ledaborbactam tested against 25 ESBL-positive isolates of *Enterobacterales* (11), while Mendes et al. reported an MIC_90_ of 0.12 μg/mL for ceftibuten-ledaborbactam tested against 50 ESBL-positive isolates of *Enterobacterales* (13). The application of EUCAST susceptibility breakpoints for ceftibuten (≤1 μg/mL) to the data set reported by Mendes et al. showed that 98.0% of ESBL-positive isolates were inhibited by ceftibuten-ledaborbactam (13).

We also observed that 85.9% of KPC-positive and 82.9% of OXA-48 group-positive isolates were inhibited by ceftibuten-ledaborbactam at ≤1 μg/mL (Table 2). Previously, Chatwin et al. reported MIC_90_s of 1 μg/mL for ceftibuten-ledaborbactam tested against both KPC- (*n *= 25), and OXA-48-carrying (*n *= 25) isolates of *Enterobacterales* (11). Mendes et al. reported ceftibuten-ledaborbactam MIC_90_s of 0.5 and 1 μg/mL, respectively, for 50 KPC-positive and 52 OXA-48-like-positive isolates of *Enterobacterales*; 92.0% of KPC-positive isolates and 94.0% of OXA-48-like-positive isolates were inhibited by ceftibuten-ledaborbactam at a concentration of ≤1 μg/mL (13). Another earlier study showed that although ceftibuten-ledaborbactam was active against OXA-48/OXA-48-like producers in isolation or in isolates also producing an ESBL, MICs were elevated against isolates coproducing OXA-48 group and CMY or OXA-48 group and DHA (12). Overexpression of an ESBL (CTX-M-15) or KPC (KPC-2 or KPC-3) in isogenic strains of E. coli did not significantly increase ceftibuten-ledaborbactam MICs, while the overexpression of an AmpC β-lactamase (P99 or CMY-42) increased MICs from 0.25 μg/mL (control) to 4 μg/mL (20). Expression levels of certain β-lactamases may account for the reduced activity of ceftibuten-ledaborbactam in some isolates, with the caveat that analysis of gene expression would be needed to definitively identify the reason for the discordance. Another explanation is that ceftibuten is an excellent substrate for hydrolysis by class C enzymes, such that even though ledaborbactam is highly active against them, reducing ceftibuten MICs by, for example, 256- or 512-fold, it may not be sufficient to lower the MIC of the combination to below 1 μg/mL for all isolates.

Ceftibuten activity was not restored by ledaborbactam in MBL-producing isolates, confirming an earlier report (12), and this is consistent with the spectrum of inhibitory activity of ledaborbactam as determined in biochemical assays (7, 9). Currently there are no approved β-lactam–β-lactamase inhibitor combinations that are active against MBL-producing isolates.

Our study has at least three limitations. First, an examination of non-β-lactamase-mediated resistance mechanisms (e.g., porin mutation/expression and efflux pump expression), which are known to affect the activity of cephalosporins, including ceftibuten, and β-lactam–β-lactamase inhibitor combinations (21), was outside the scope of this investigation. Second, no data are included regarding isolate background, including clinical syndrome and underlying host comorbidities, or associated clinical or microbiological outcomes in patients from which the isolates were obtained. Third, our modified definition of an ESBL phenotype, without confirmatory testing with clavulanic acid (15), may have resulted in the inclusion of a limited number of isolates with other or additional mechanisms of β-lactam resistance (e.g., acquired AmpC, efflux, porin changes, and penicillin-binding protein [PBP] changes). Fourth, not all presumptive ESBL-positive isolates were subjected to molecular testing.

Based on results from the current study, ceftibuten-ledaborbactam etzadroxil appears to have potential as an oral treatment option for complicated urinary tract infections caused by serine β-lactamase-expressing *Enterobacterales* (ESBL, KPC, and OXA-48 group) for which there are currently few oral treatment options available and a global medical need exists. Ceftibuten-ledaborbactam exhibited potent *in vitro* activity against isolates that were not susceptible to current, frequently prescribed oral agents (trimethoprim-sulfamethoxazole, amoxicillin-clavulanate, levofloxacin, and nitrofurantoin) or to parenteral agents (ceftazidime, cefepime, and carbapenems). Further clinical development of ceftibuten-ledaborbactam etzadroxil is warranted as the first oral agent to address the unmet need for treatments for increasingly common WHO priority pathogens.

## MATERIALS AND METHODS

### Bacterial isolates.

*Enterobacterales* isolates (*n *= 3,889) collected by 229 hospital laboratory sites in 52 countries in seven global regions from 2018 to 2020 (Table S4) and maintained by IHMA in their frozen (−80°C) global surveillance isolate collection were tested in this study. Isolates were identified to species level at IHMA by matrix-assisted laser desorption ionization–time of flight (MALDI-TOF) mass spectrometry (Bruker Daltonics, Bremen, Germany) to confirm their identities (Table S5). The top five species tested were E. coli (36.1%), K. pneumoniae (28.7%), K. oxytoca (4.7%), P. mirabilis (4.6%), and Citrobacter freundii (3.8%). Isolate sources included bloodstream infections (823 [21.2%]), intra-abdominal infections (409 [10.5%]), respiratory tract infections (998 [25.7%]), skin and soft tissue infections (449 [11.5%]), and urinary tract infections (1,210 [31.1%]) (Table S1). Isolates were predominantly from 2020 (3,332 isolates; 85.7% of isolates tested); 397 isolates (10.2%) were from 2018, and 160 isolates (4.1%) were from 2019 (Table S5).

### Antimicrobial susceptibility testing.

MICs were determined using the CLSI reference broth microdilution method (22). Broth microdilution panels were prepared at IHMA using cation-adjusted Mueller-Hinton broth (CAMHB) (Becton, Dickinson, Sparks, MD) and stored at −80°C until the day of testing. CAMHB with TES [*N*-tris(hydroxymethyl)methyl-2-aminoethanesulfonic acid; TREK Diagnostic Systems, Independence, OH] was used for inoculum preparation. Tryptic soy agar (TSA) plates containing 5% sheep blood (Liofilchem, Waltham, MA) were used to subculture isolates.

Ledaborbactam was provided by Venatorx Pharmaceuticals. Other antimicrobial agents were purchased from commercial sources. Ledaborbactam was dissolved in dimethyl sulfoxide (DMSO) to make initial solutions with concentrations of 5,120 μg/mL; these solutions were diluted 1:10 in sterile water to create 512-μg/mL stock solutions. MICs of ceftibuten-ledaborbactam were determined at a fixed concentration of 4 μg/mL for ledaborbactam (7).

Quality control testing was performed each day that clinical isolates were tested (15, 22). Ceftibuten MICs were within the CLSI-approved quality control ranges for E. coli NCTC 13353 (0.03 to 0.25 μg/mL) and K. pneumoniae ATCC BAA-2814 (0.5 to 2 μg/mL) (CLSI 2021 Winter AST Plenary 05A QCWG report draft 4 [https://clsi.org/meetings/ast-file-resources/]). We anticipate publication of consensus reference quality control ranges and strains for broth microdilution testing of ceftibuten-ledaborbactam in the 33rd edition of CLSI M100 in January 2023.

MICs were interpreted using CLSI (15) and EUCAST (17) breakpoints published in 2022. Amoxicillin-clavulanate was tested in a 2:1 ratio (15), and therefore, MICs could not be interpreted using EUCAST breakpoints, which require clavulanate to be tested at a fixed concentration of 2 μg/mL (17). For cefazolin, MICs were interpreted using CLSI parenteral breakpoints (susceptible, ≤2 μg/mL; intermediate, 4 μg/mL; resistant, ≥8 μg/mL). CLSI publishes investigational MIC breakpoints for ceftibuten (susceptible, ≤8 μg/mL; intermediate, 16 μg/mL; resistant, ≥32 μg/mL) for testing and reporting of *Enterobacterales* urinary tract isolates only (15). EUCAST publishes MIC breakpoints for ceftibuten (susceptible, ≤1 μg/mL; resistant, >1 μg/mL) for *Enterobacterales* infections originating from the urinary tract that are also based on an oral once-daily 400-mg dose (17). While the clinical dose and dose regimen for ceftibuten-ledaborbactam remain to be determined, ceftibuten exposures reflecting twice the approved ceftibuten-only dose of 400 mg once per day were effective in a translational neutropenic mouse model of thigh infection with ceftibuten-resistant *Enterobacterales* when combined with ledaborbactam (23). For comparative purposes, ceftibuten-ledaborbactam MICs were interpreted using two provisional susceptibility breakpoints, ≤1 μg/mL and ≤0.5 μg/mL. Tebipenem MICs were interpreted using a provisional susceptibility breakpoint of ≤0.12 μg/mL (16).

MDR phenotypes were based on the criteria of Magiorakos et al. (14) and included isolates that were nonsusceptible (intermediate or resistant) to at least one agent in three or more of the following antimicrobial categories: fluoroquinolones (levofloxacin), non-extended-spectrum cephalosporins (i.e., first- and second-generation cephalosporins [cefazolin]), extended-spectrum cephalosporins (ceftazidime and/or cefepime), aminoglycosides (gentamicin), penicillins plus β-lactamase inhibitors (amoxicillin-clavulanate), antipseudomonal penicillins plus β-lactamase inhibitors (piperacillin-tazobactam), carbapenems (meropenem and/or imipenem), and folate pathway inhibitors (trimethoprim-sulfamethoxazole). Species known to be intrinsically resistant to specific antimicrobial agents or categories were excluded when determining MDR status (14). ESBL phenotype screening criteria were modified from those published by CLSI (15). For the purpose of this study, an ESBL-positive phenotype was assigned to isolates of E. coli, K. pneumoniae, K. oxytoca, and P. mirabilis with ceftazidime MICs of ≥2 μg/mL and meropenem MICs of ≤1 μg/mL. Carbapenem-nonsusceptible *Enterobacterales* isolates were defined as those with meropenem MICs of ≥2 μg/mL (all isolates) and/or imipenem MICs of ≥2 μg/mL (non-*Proteeae* isolates).

### Molecular studies.

All isolates with ceftibuten-ledaborbactam MICs of ≥2 μg/mL were interrogated by either whole-genome sequencing (*n *= 99) or PCR followed by Sanger sequencing (*n *= 62) for β-lactamase genes. An additional set of isolates with ceftibuten-ledaborbactam MICs of <2 μg/mL were also examined by PCR followed by Sanger sequencing (*n *= 275) and whole-genome sequencing (*n *= 4). This additional set of isolates included isolates that had meropenem MICs of ≥4 μg/mL, cefepime and/or ceftazidime MICs of ≥2 μg/mL, and/or cefepime-taniborbactam MICs of ≥16 μg/mL (based on previous testing of the isolates). The molecular methods used are described in detail in Appendix S1 in the supplemental material (also, see references 24 and 25).
