# Supplementary material for: Ceftibuten-Ledaborbactam Activity against Multidrug-Resistant and Extended-Spectrum-β-Lactamase-Positive Clinical Isolates of Enterobacterales from a 2018–2020 Global Surveillance Collection
Source: Antimicrob Agents Chemother. 2022 Oct 26;66(11):e00934-22. doi: 10.1128/aac.00934-22 (PMC9664860; doi:10.1128/aac.00934-22)
Supplement: Supplemental file 1 — Supplemental material. Download aac.00934-22-s0001.pdf, PDF file, 0.2 MB [file aac.00934-22-s0001.pdf]

SUPPLEMENTAL TABLE S1 Summary of the *in vitro* activities of ceftibuten and ceftibuten-ledaborbactam against *Enterobacterales* isolates stratified by global region and infection source

	Ceftibuten MIC (µg/ml)		Ceftibuten-ledaborbactam MIC (µg/ml)		Cumulative % of isolates inhibited at ceftibuten-ledaborbactam MIC	
Global region[a]/infection source (*n*)	MIC_{50}	MIC_{90}	MIC_{50}	MIC_{90}	≤0.5 µg/ml	≤1 µg/ml
All isolates (3,889)	0.25	32	0.06	0.25	94.4	95.9
North America (1,615)	0.25	16	0.06	0.25	95.6	96.7
Europe (1,289)	0.25	32	0.06	0.25	95.0	96.8
Latin America (472)	0.25	32	0.06	0.25	93.7	94.9
Asia (306)	0.5	>32	0.06	1	87.9	90.5
Bloodstream infection (823)	0.25	32	0.06	0.25	95.5	96.8
Intraabdominal infection (409)	0.25	32	0.06	0.25	93.9	95.6
Respiratory tract infection (998)	0.25	>32	0.06	0.5	92.9	94.8
Skin and soft tissue infection (449)	0.12	16	0.03	0.25	94.9	96.7
Urinary tract infection (1,210)	0.25	32	0.06	0.25	94.9	95.9

[a] Excluded global regions with <100 isolates (Africa, Middle East and South Pacific).

SUPPLEMENTAL TABLE S2 *In vitro* activity of ceftibuten-ledaborbactam and comparator agents against 1,210 urinary tract infection isolates of *Enterobacterales*

Agent dosage form	μg/ml			MIC interpretation – CLSI			MIC interpretation – EUCAST	
Antimicrobial agent	MIC_{50}	MIC_{90}	MIC range	% susceptible	% intermediate	% resistant	% susceptible	% resistant
Oral or oral/intravenous agent								
Ceftibuten-ledaborbactam[a,b]	0.06	0.25	≤0.016->32	94.9/95.9	NA[c]	5.1/4.1	94.9/95.9	5.1/4.1
Ceftibuten[d]	0.25	32	≤0.06->32	86.0	3.6	10.5	73.1	26.9
Amoxicillin-clavulanate[e]	4	32	≤2->32	73.4	10.4	16.2	NA	NA
Cefixime	0.5	>8	≤0.06->8	66.4	3.6	30.0	66.4	33.6
Levofloxacin	0.12	>8	≤0.004->8	65.2	4.5	30.3	65.2	30.3
Nitrofurantoin	16	>128	≤2->128	63.5	14.7	21.8	78.2	21.8
Tebipenem[f]	0.03	0.25	0.008->4	87.3	NA	12.7	87.3	12.7
Trimethoprim-sulfamethoxazole	≤0.25	>4	≤0.25->4	64.7	NA	35.3	64.7	35.3
Intravenous agent only								
Cefazolin[g]	4	>32	≤0.5->32	41.3	9.7	49.1	UTD[h]	49.0
Cefepime[i]	≤0.25	>16	≤0.25->16	83.3	NA	16.7	76.7	19.4
Ceftazidime	0.5	>16	≤0.03->16	76.0	2.6	21.4	71.6	24.0
Imipenem-relebactam	0.12	1	≤0.03->8	91.0	5.8	3.2	96.8	3.2
Imipenem	0.12	2	≤0.03->16	89.2	5.7	5.1	94.9	3.0
Meropenem	0.03	0.12	≤0.004->64	96.8	0.2	3.0	97.1	2.1

[a] Ledaborbactam was tested at a fixed concentration of 4 μg/ml in combination with doubling dilutions of ceftibuten.
[b] Provisional susceptible MIC breakpoints of ≤0.5 μg/ml/≤1 μg/ml and provisional resistant MIC breakpoints of ≥1 μg/ml/2 μg/ml applied for ceftibuten-ledaborbactam.
[c] NA, not applicable.
[d] CLSI publishes investigational MIC breakpoints for ceftibuten (susceptible, ≤8 μg/ml; intermediate 16 μg/ml; resistant, ≥32 μg/ml) for testing and reporting of urinary tract isolates only (15). EUCAST publishes MIC breakpoints for ceftibuten (susceptible, ≤1 μg/ml; resistant, >1 μg/ml) for infections originating from the urinary tract (17).
[e] For amoxicillin-clavulanate MICs, the EUCAST uncomplicated urinary tract infection only breakpoints were used (susceptible, ≤32 μg/ml; resistant, >32 μg/ml).
[f] Provisional susceptible breakpoint of ≤0.12 μg/ml applied for tebipenem (16).
[g] For cefazolin MICs, the CLSI parenteral breakpoints were used (susceptible, ≤2 μg/ml; intermediate 4 μg/ml; resistant, ≥8 μg/ml).
[h] UTD, unable to determine because the cefazolin concentration range tested did not encompass the susceptible breakpoint.
[i] Percentage determined using the cefepime susceptible-dose dependent breakpoint. CLSI does not define an intermediate MIC breakpoint for cefepime tested against *Enterobacterales*.

SUPPLEMENTAL TABLE S3 Summary of the *in vitro* activities of ceftibuten and ceftibuten-ledaborbactam against *Enterobacterales* isolates stratified by species

	Ceftibuten MIC (µg/ml)		Ceftibuten-ledaborbactam MIC (µg/ml)		Cumulative % of isolates inhibited at ceftibuten-ledaborbactam MIC	
Enterobacterales group/species (*n*)	MIC_{50}	MIC_{90}	MIC_{50}	MIC_{90}	≤0.5 µg/ml	≤1 µg/ml
All isolates (3,889)	0.25	32	0.06	0.25	94.4	95.9
Citrobacter freundii complex (259)	1	>32	0.03	0.5	92.3	95.8
Enterobacter cloacae complex (222)	2	>32	0.06	4	77.5	82.0
Escherichia coli (1,405)	0.25	16	0.06	0.12	98.2	98.4
Klebsiella spp. (1,392)	≤0.06	>32	0.03	0.5	92.2	94.3
Proteeae (435)	≤0.06	8	0.03	0.06	97.9	98.2
Serratia spp. (176)	0.25	2	0.12	0.25	97.7	99.4

SUPPLEMENTAL TABLE S4 *Enterobacterales* isolate counts by global region and country

Global region<br>Country	No. of isolates	% of total no. of isolates
Africa	80	2.1%
Morocco	20	0.5%
Nigeria	21	0.5%
South Africa	39	1.0%
Asia	306	7.9%
Hong Kong	7	0.2%
India	59	1.5%
Japan	38	1.0%
Korea, South	94	2.3%
Malaysia	9	0.2%
Taiwan	40	1.0%
Thailand	59	1.5%
Europe	1,289	33.1%
Belgium	123	3.2%
Croatia	37	1.0%
Czech Republic	55	1.4%
Denmark	12	0.3%
Finland	2	0.1%
France	129	3.3%
Germany	137	3.5%
Greece	58	1.5%
Hungary	44	1.1%
Ireland	9	0.2%
Italy	146	3.8%
Latvia	4	0.1%
Lithuania	38	1.0%
Netherlands	18	0.5%
Poland	44	1.1%
Portugal	33	0.8%
Romania	28	0.7%
Russia	29	0.7%
Spain	149	3.8%
Sweden	13	0.3%
Switzerland	30	0.8%
Turkey	57	1.5%
Ukraine	21	0.5%
United Kingdom	73	1.9%
Latin America	472	12.1%
Argentina	45	1.2%
Brazil	46	1.2%
Chile	46	1.2%
Colombia	82	2.1%
Costa Rica	16	0.4%
Dominican Republic	21	0.5%
Guatemala	26	0.7%
Mexico	108	2.8%
Panama	30	0.8%
Venezuela	52	1.3%
Middle East	74	1.9%
Israel	51	1.3%
Kuwait	15	0.4%
Saudi Arabia	8	0.2%
North America	1,615	41.5%
Canada	147	3.8%
United States	1,468	37.7%
South Pacific	53	1.4%
Australia	40	1.0%
New Zealand	1	<0.1%
Philippines	12	0.3%
Grand Total	3,889	100%

SUPPLEMENTAL TABLE S5 Isolate counts by *Enterobacterales* species, overall and by year of collection

Species of *Enterobacterales*	No. of isolates (% of total no. of isolates)	Year collected		
		2018	2019	2020
Citrobacter amalonaticus	4 (0.1)			4
Citrobacter braakii	14 (0.4)			14
Citrobacter farmeri	2 (<0.1)			2
Citrobacter freundii	149 (3.8)	6	2	141
Citrobacter koseri	84 (2.2)	1		83
Citrobacter sedlakii	2 (<0.1)			2
Citrobacter sp.	3 (0.1)			3
Citrobacter youngae	1 (<0.1)			1
Enterobacter asburiae	10 (0.3)			10
Enterobacter bugandensis	21 (0.5)			21
Enterobacter cloacae	138 (3.5)	16	10	112
E. cloacae complex, unable to speciate	12 (0.3)			12
Enterobacter kobei	1 (<0.1)			1
Enterobacter ludwigii	1 (<0.1)			1
Enterobacter sp.	20 (0.5)			22
Enterobacter xiangfangensis	19 (0.5)			19
Escherichia coli	1,405 (36.1)	278	113	1,014
Klebsiella aerogenes	90 (2.3%)			90
Klebsiella oxytoca	183 (4.7)	3	2	178
Klebsiella pneumoniae	1,118 (28.7)	56	24	1,038
Klebsiella variicola	1 (<0.1)			1
Morganella morganii	88 (2.3)	4	1	83
Proteus mirabilis	181 (4.6)	25	6	150
Proteus vulgaris	77 (2.0)			77
Providencia alcalifaciens	1 (<0.1)			1
Providencia rettgeri	43 (1.1)		1	42
Providencia stuartii	45 (1.2)	4	1	40
Serratia liquefaciens	2 (0.1)			2
Serratia marcescens	101 (2.6)	4		97
Serratia sp.	68 (1.7)			68
Serratia ureilytica	5 (0.1)			5
Total	3,889 (100)	397	160	3,332

SUPPLEMENTAL APPENDIX S1 Description of the molecular methods used to characterize selected isolates of *Enterobacterales*

Whole genome sequencing. Cells were pelleted from 3 ml liquid cultures grown overnight from one colony in Brain Heart Infusion broth (Sigma-Aldrich) at 37 °C with shaking. DNA was subsequently extracted using the DNeasy Ultraclean Microbial extraction kit (Qiagen). Sequencing libraries were prepared using the Illumina DNA Prep library preparation kit. Sequencing was performed on an Illumina Hiseq system using 2×150 bp paired-end reads with a target coverage depth of 100-times.

All analyses were carried out using the CLC Genomics Workbench, version 20 (Qiagen). For resistance gene identification, *de novo* assemblies of each genome were queried using the "find resistance" module, which interrogates the CGE database for resistance genes. β-lactamase genes with less than 100% sequence identity to a known nucleotide reference were translated to their deduced amino acid sequence and BLASTP searched against the Refseq database in Genbank dedicated to β-lactamase nomenclature (Bioproject 313047) in order to assign the enzyme variant.

β-lactamase characterization via PCR and Sanger sequencing. Genomic DNA for all isolates was obtained using the QIAmp® DNA Mini protocol for the QiaCube (Qiagen, Gaithersburg, MD) following the recommendations provided by the manufacturer. *Enterobacterales* isolates were screened for the presence of *bla* encoding ESBLs (TEM, SHV, CTX-M including 5 subtypes [CTX-M-1-type, that included the prevalent CTX-M-15; CTX-M-2-type, CTX-M-8-type, CTX-M-9-type and CTX-M-25-type], GES [all variants], VEB, and PER [including PER-

1-like and PER-2-like subtypes]), carbapenemases (KPC, OXA-48 group, NDM, IMP, VIM, SPM, GIM) and AmpC β-lactamases (ACC, ACT, CMY, DHA, FOX, MIR, MOX) by multiplex PCR using published primers (24, 25). Detected genes were amplified with extragenic primers and sequenced in full except for *bla*$_{TEM}$ and *bla*$_{SHV}$. These genes were screened first by limited sequencing to identify genes encoding TEM-type and SHV-type enzymes containing amino acid substitutions common to ESBLs at the following positions: SHV amino acid 146, 179, 238, 240; TEM amino acid 104, 164, 238, 240. Only *bla*$_{SHV}$ and *bla*$_{TEM}$ that encoded ESBLs were completely sequenced. Intrinsic AmpCs (ACT/MIR detected in *Enterobacter* spp., CMY detected in *Citrobacter* spp., and DHA detected in *Morganella morganii*) were confirmed but not sequenced.

For all fully sequenced β-lactamase genes, the deduced amino acid sequence was compared to available databases maintained by the NCBI (www.ncbi.nlm.nih.gov) to identify curated enzyme variants.
